# Targeting Astrogliosis in the Retrotrapezoid Nucleus: A Novel Approach to Ameliorate Respiratory Dysfunction and Alzheimer's Pathology in Mice

**DOI:** 10.14336/AD.2024.0523

**Published:** 2025-07-26

**Authors:** Zahid Iqbal, Ahmad El Hamamy, Ngoc Mai Le, Arya Ranjan, YuXing Zhang, Li Qi, Bharti Manwani, Chunfeng Tan, Louise D. McCullough, Jun Li

**Affiliations:** The University of Texas Health Science Center at Houston and the McGovern Medical School, Houston TX, 77030, USA

**Keywords:** Alzheimer's disease, Disordered breathing, TGFβR II, RTN

## Abstract

Alzheimer's disease (AD), a leading cause of dementia, is associated with significant respiratory dysfunctions. Our study explores the role of astrogliosis in the brainstem retrotrapezoid nucleus (RTN), a key breathing regulatory center, and its impact on breathing control and AD pathology in mice. Using Tg-2576 AD and wild-type mice, we investigated the effect of silencing the transforming growth factor-beta receptor II (TGFβR II) in the RTN. We performed behavioral tests, including the Barnes maze and novel object recognition test, along with whole-body plethysmography to assess breathing disorders. Our results showed that AD mice exhibited increased apneas and cognitive impairment, which were significantly mitigated following TGFβR II gene silencing. Immunohistochemistry revealed elevated levels of GFAP and TGFβR II in the RTN of AD mice, which were reduced post-gene silencing, alongside a decrease in amyloid-beta expression in the cortex and hippocampus. These findings suggest that targeting astrogliosis and improving respiratory control may offer a novel therapeutic avenue for managing Alzheimer's disease. Our study provides the first mechanistic insights into how TGFβ signaling influences both respiratory control and AD pathogenesis, highlighting the potential benefits of stabilizing breathing patterns in AD treatment.

Alzheimer’s disease (AD), the seventh leading cause of death, is the most common type of dementia, comprising 60-70% of the dementia cases [1]. The disease is characterized by the accumulation and deposition of β-amyloid protein in the parenchyma of brain, leading to the formation of amyloid plaques [2-4]. Apnea is an independent risk factor for the development of cognitive dysfunction and dementia. Our group has previously shown breathing disorder and its correlation of cognitive impairment in aged stroke mice [5]. In patients with AD, disordered breathing also occurs frequently [6]. In cognitively impaired populations, the most severely demented patients had the worst sleep disordered breathing. Changes in sleep precede the onset of cognitive symptoms in patients with AD, and a strong association exists between disrupted sleep and the development of AD [7]. Respiratory rhythm is generated by neurons in the pre-Bötzinger complex (pre-BötC) that control inspiration, and a subset of neurons located in the parafacial respiratory group that regulate active expiration. The output of these neurons is relayed to respiratory motor neurons to influence rate and depth of breathing. Central and peripheral chemoreceptors regulate respiratory rhythm based on changes in CO_2_/H^+^ and O_2_. The RTN (retrotrapezoid nucleus) contains central chemoreceptors in the ventral surface of the medulla oblongata (VMS) that monitor levels of CO_2_/H^+^ in the brain, sending projections to the pre-Botzinger’s complex [8]. However, it is unknown how breathing control is impaired in AD, we used Tg-2576 mouse model to investigate the underlying cause with a focus on retrotrapezoid nucleus in brain stem breathing center. The Tg-2576 mouse model is widely used in Alzheimer's disease research. This model overexpresses the human amyloid precursor protein (APP) with the Swedish mutation (KM670/671NL) [9]. We hypothesized that the astrogliosis at RTN is the major driver of the breathing impairment in AD, and we further tested whether inhibiting the signaling of TGFβ, a key inducer of astrogliosis, can reduce gliosis, improve breathing control and reduce AD pathology.

**Figure 1.**
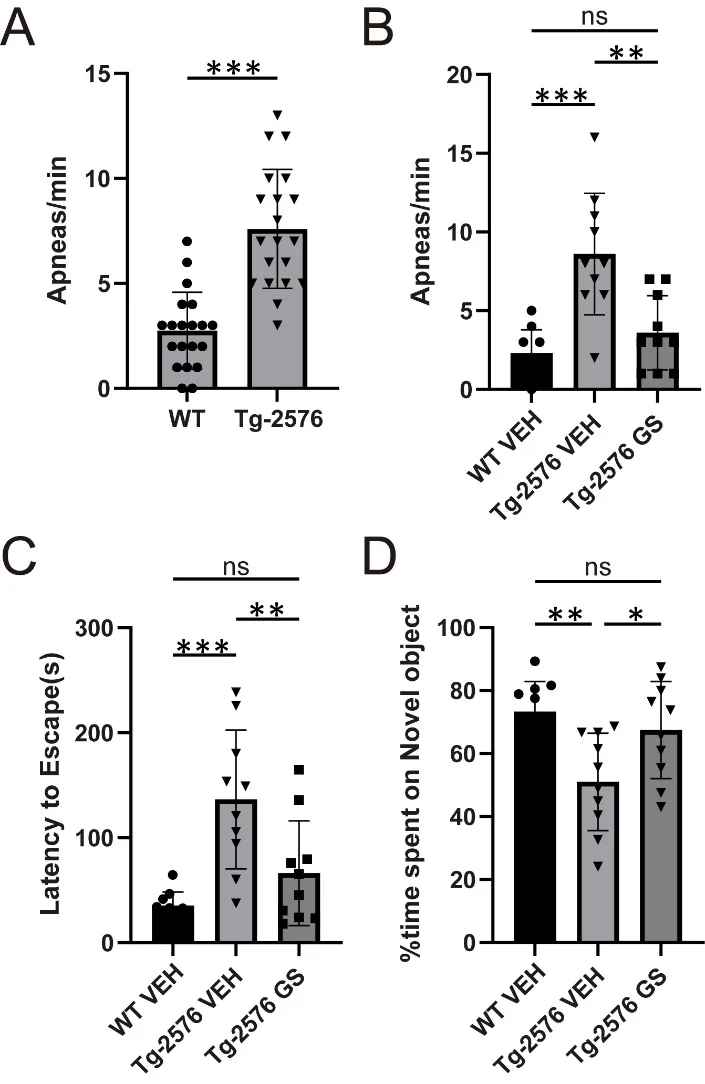
**Behavior function and breathing assays in WT and Tg-2576 mice after TGFβR II GS**. (**A**) Baseline testing showed TG-2576 mice had increased number of apneas per minute, *P*<0.01. n=20/group. (**B**) The number of apneas was markedly decreased after TGFβR II GS in AD mice, *P*<0.01. n=10/group. (**C**) Barnes maze showed decreased escape time for the AD mice with TGFβR II GS, *P*<0.01. n=10/group. (**D**) AD mice with TGFβR II GS had improved recognition of a novel object in the cognitive assessment by NORT, *P*<0.05. n=10/group. One-way ANOVA test was applied for these functional assays.

Tg-2576 (AD) and wild type (WT) 15 months old mice were procured, all the animal experiments were performed in accordance with the guidelines of the National Institute of Health for the handling and care, and the animal use for the experiments was approved by the University of Texas Health Science Center Houston Institutional Animal Care and Use Committee and reported according to the guidelines of ARRIVE (Animal Research: Reporting of *In Vivo* Experiments). After baseline behavior and breathing testing, Tg-2576 mice were randomly assigned to two subgroups; Vehicle control (Tg-2576 VEH) and Gene Silenced (Tg-2576 GS). WT and Tg-2576 Vehicle control groups received Lenti-ONE-GFP-gfa2-GFP as control virus, while Tg-2576 GS group was administered with lentiviral particles encoding gfa2-gRNA-miR124T/CMV-Cas9 (astrocytic specific, provided by GEG-Tech France). The lentivirus particles were injected into the retrotrapezoid nucleus (RTN) bilaterally. The lentivirus with a concentration of 2x10^9^ TU/ml was delivered using a Hamilton needle at coordinates 1.0 mm lateral to the midline, 5.6 mm caudal to bregma, and 5.6-5.7 mm ventral to the pial surface of the cerebellum bilaterally, as described earlier [10, 11]. A total volume of 300 nL per hemisphere was administered at a rate of 300 nL/min. To ensure precise targeting of the compact RTN, we utilized an ultra-precise digital stereotaxic device (Stoelting). This technology enhances the accuracy of our region-specific targeting. Furthermore, we examined the TGFβR II expression in adjacent regions, such as the NTS region in the brainstem, and found no effects outside of the RTN.

Six weeks post-injections, cognitive assessment was done by Barnes maze and novel object recognition test (NORT). Barnes maze was performed as previously described [12, 13]. Briefly, mice were trained to find an escape box placed under one of the holes in a flat circular maze. Mice completed 4 trials per day for 3 consecutive days during the acquisition phase and each mouse had 5 minutes to explore the maze. On the fourth day, a single probe trial was carried out for the mice to search the maze and latency to find the escape hole was recorded. NORT was conducted to test non-hippocampal mediated cognition as described earlier [14]. Mice were placed in open field for 20 mins to acclimate to the environment on first day. On the second day, two identical objects were placed in the open field in opposite quadrants, while one of the objects was replaced by a novel object on the third day. For all trials, testing was stopped after each mouse attained a total exploration time of 30 seconds between the two objects. The trials were recorded and analyzed by EthoVision, and the % time spent on novel object was calculated. As a respiratory parameter, apnea count was measured by using whole body plethysmography in mice as we previously performed [5]. Briefly, mice were acclimated for 30 minutes before recording the parameters. A dual criterion approach was adopted for the quantification of apneic events. First, a pause in breathing needs to be lasting for more than twice of the duration of average breathing time to be considered as an apneic event, according to well-established criteria [5]. Secondly, a breathing pause needs to be lasting for at least 0.5 seconds to be considered as an apnea [15]. Apneas per minute was obtained by calculating the average apnea number during the recording period of 20 minutes.

**Figure 2.**
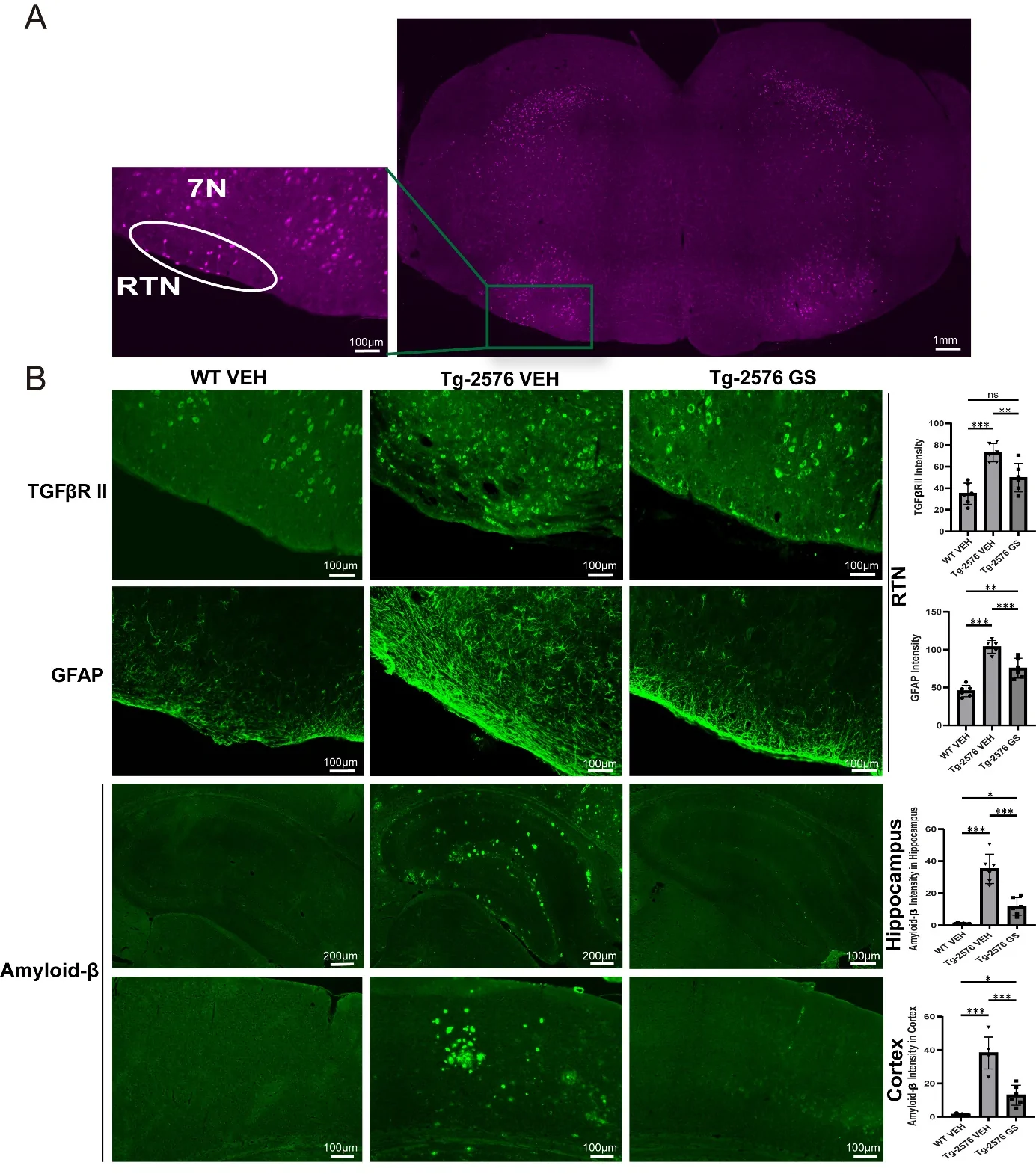
**TGFβR II GS in Tg-2576 brainstem RTN ameliorated AD pathology**. (**A**) RTN area in brain stem was visualized by phox 2B staining (ventral surface area with brighter phox 2B staining and below 7N. (**B**) TGFβR II GS was confirmed in the RTN area and GFAP staining showed decreased gliosis after the gene silencing in the area. Amyloid-β level was significantly decreased in the hippocampus and cortex of the mice. ImageJ software was used to quantify the protein levels and One-way ANOVA test was applied, *P*<0.05. n=6/group.

After functional testing, mice were sacrificed for the collection of brains which were processed for optimal cutting temperature (OCT) embedding and later sliced into 30 µm thick coronal sections. Immunohistochemistry (IHC) staining was performed for TGFβR II (1:200, ab186838), GFAP (1:200, Cat# 12389S) and Aβ (1:1000, Cat# ab201060) expression level. Brain stem RTN was visualized/identified by phox 2B (1:200, Cat# AF4940) staining and its known anatomical relation with the 7^th^ nucleus (7N) cluster as shown in Figure 2A [16, 17]. Appropriate secondary antibodies (1:2000, anti-Rabbit Cat# ab150073 and anti-Goat Cat# ab150132) were used. To rule out non-specific antibody binding, controls for primary and secondary antibodies were processed without primary and secondary antibodies respectively. For each brain, three brainstem sections (at 30 µm interval) for RTN, and three brain sections (at 0.5 mm interval) for cortex and hippocampus were separately imaged using a 20x lens with Leica Thunder Imager DMi8 (Leica, Heerbrugg, Switzerland). ImageJ software version 1.54j was used for the expression quantification. After converting images to grayscale, background subtraction and thresholding was applied to segment positive staining. Fluorescence intensity was measured as mean gray value and normalized to isotype control. Images with quantification value closest to the median value of the respective group are given as the representative images. For all statistical analysis, GraphPad Prism 10 was used, and all the data are presented as Mean±SEM [18]. The Shapiro-Wilk test was performed to assess the normality of the data, and One-way ANOVA test was used for the normal data analysis. Investigators were blinded by behavioral tests and all kinds of data analysis. We found the AD mice exhibited disordered breathing as they had higher number of apneas per minute as compared to WT control group (*P*<0.01; Fig. 1A). Silencing of the TGFβR II reduced the number of apneas in AD mice 6 weeks post-injections (*P*<0.01, Fig. 1B). AD mice with the receptor silenced exhibited marked improvement in cognitive behavior as they required less time to escape on Barnes maze (*P*<0.01, Fig. 1C). In NORT test, TGFβR II GS improved AD mice performance in recognizing the new object (*P*<0.05, Fig. 1D). We performed IHC staining of the mice brains to study the pathological changes in AD mice and the effect of the receptor gene silencing in AD mice. We found that AD mice showed increased both GFAP and TGFβR II levels at RTN. Vector mediated gene silencing reduced the TGFβR II level in RTN region of the mice brainstem and it also decreased GFAP expression level in the RTN (Fig. 2B). Interestingly, we also observed decreased levels of Aβ expression in cortex and hippocampus after gene silencing.

Our data suggested that astrogliosis in chemo-receptor regions leads to breathing dysfunction in AD mice. Our study is the first report of astrogliosis in the RTN and its causal role in breathing disorder in mice with AD. Astrogliosis, characterized by increased GFAP expression, is a hallmark of AD pathology. For instance, one paper showed that the astrogliosis in AD mouse model was associated with chronic intermittent hypoxia [19]. Astrogliosis in the RTN may disrupt breathing via enhanced basement membrane fibrosis or as a physical barrier, which could inhibit both neuronal and astrocytic detection of CO_2_/H^+^. We found that by stabilizing breathing control, Aβ burden is reduced in the AD brain. Breathing disorders that produce sleep interruption and intermittent hypoxia lower cerebral spinal fluid (CSF) Aβ42 levels (due to poorer glymphatic clearance) [20]. Other studies suggest that oxidative stress induced by chronic intermittent hypoxia increases generation of Aβ [21]. Our work demonstrated that breathing disorder/apnea contributes to an increase in brain Aβ burden and worsens the progressive cognitive decline in AD patients.

Literature suggests a contributory role for TGFβ in cerebrovascular amyloidosis. TGFβ increases in AD brains of patients and the expression of TGFβ1 in astrocytes in mice resulted in perivascular astrogliosis and age-related deposition of amyloid around cerebral blood vessels mirroring CAA pathology [22]. Importantly, vessels from these mice had prominent perivascular astrogliosis [22]. TGFβ can regulate astrocyte reactivity by directly activating GFAP promoter [23], by triggering different regulators of reactive astrogliosis, including Jak-Stat, NF-κB and MAP-KKK [24] or by modulating its downstream target genes [25]. A study in AD mouse model (APP/PS1) showed that astrocytic-specific knockout of ApoE improved the cognitive behavior by markedly reducing astrogliosis and Aβ production, while TGFβ overexpression abolished the protective role of ApoE knockout, signifying that the knockout effect was TGFβ dependent [26].

While the Tg-2576 model provides valuable insights into amyloid-β pathology and its role in cognitive impairment, there are several limitations to consider: First, cognitive impairment in Tg-2576 mice develops at 9-10 months of age, which is mid-age for mice and earlier than the typical onset of AD in humans. Second, the Tg-2576 model exhibits very limited neuronal loss. In contrast, human AD is characterized by significant neuronal dropout, particularly in regions such as the hippocampus and cortex [27]. Third, since the RTN area is too small and our target is only astrocytic TGFβR II, we could confirm the silencing by IHC staining only. Despite these limitations, the Tg-2576 model remains a very useful tool for studying the amyloid-related aspects of AD pathology. The data reported from research using this model are interpreted with these limitations in mind, and the findings contribute to a broader understanding of the disease. Further studies should be aimed to explore the specific mechanism of how TGFβ signaling affects astrogliosis in different mouse models before investigating the TGFβ signaling inhibition effect in human patients. It is very important to test the effects of our treatment in different dementia models to ensure the robustness and applicability of our results. Respiratory disorders are very common in dementia patients, and it is likely that other AD mouse models may also develop breathing disorders similar to those observed in our Tg-2576 mice. Our future directions include examining the respiratory function of other dementia mouse models to determine if breathing impairments and brainstem gliosis are present. Should these conditions be observed, we will proceed with silencing the receptor in these models to evaluate whether our findings are generalizable.

Our work is the first report that provided a mechanistic insight into how TGFβ signaling influences breathing control and AD pathogenesis.

In summary, our study revealed breathing disorder and pathological changes in the breathing control center in AD mice. We showed that the silencing of TGFβR II in the RTN improved both the cognitive and breathing phenotypes in AD-model mice. Our work suggests that improving respiratory control and reducing apnea in AD may be a potential treatment for the disease.
